# Associations of IL-4, IL-6, and IL-12 levels in peripheral blood with lung function, cellular immune function, and quality of life in children with moderate-to-severe asthma

**DOI:** 10.1097/MD.0000000000006265

**Published:** 2017-03-24

**Authors:** Ai-Hua Cui, Jing Zhao, Shu-Xiang Liu, Ying-Shuang Hao

**Affiliations:** Department of Pediatrics, Liaocheng People's Hospital, Liaocheng, Shandong Province, PR China.

**Keywords:** asthma, cellular immune function, interleukin-4, interleukin-6, interleukin-12 lung function, QOL

## Abstract

**Background:**

Pediatric asthma has gained increasing concerns with poorly understood pathogenesis. The purpose of this study was to explore the associations of interleukin-4 (IL-4), IL-6, and IL-12 levels in peripheral blood (PB) with lung function, cellular immune function, and children's quality of life (QOL) with moderate-to-severe asthma.

**Methods:**

A total of 1158 children with moderate-to-severe asthma (the experimental group) and 1075 healthy children (the control group) were recruited for our study. Enzyme-linked immunosorbent assay was used to detect IL-4, IL-6, and IL-12 levels. T lymphocytes were detected by alkaline phosphatase antialkaline phosphatase, and erythrocyte immune was measured by red blood cell C 3b receptor (RBC-C3bR) rosette-forming test. The forced expiratory volume in 1 second (FEV1) and peak expiratory flow (PEF) were detected, after which FEV1/forced vital capacity (FVC) was calculated before and after treatment. PedsQL3.0 was used to measure the effect of asthma on QOL of children, and the correlation between IL-4, IL-6, and IL-12 levels and the lung function and QOL was measured. Logistic regression analysis was applied to detect related factors of moderate-to-severe asthma of children.

**Results:**

After treatment, the decreased IL-4 and IL-6 levels and increased IL-12 level were revealed in the experimental group. The cellular immune function's disorder was significantly decreased, and an elevated CD_3_, CD_4_, CD_8_, and declined CD_4_/CD_8_ level was performed in T lymphocytes. RBC-C3bR was increased, and red blood cell immune complex (RBC-IC) was reduced in erythrocyte immune in comparison with those before treatment. Lung function parameters all increased. After treatment, the symptoms of asthma in children reduced with scores of increased QOL. IL-4 was positively related to RBC-IC, but negatively associated with the QOL score. IL-6 showed negative connection with CD_4_/CD_8_, RBC-C3bR, FEV1/FVC, and QOL score, and had positive connection with PEF. In addition, IL-12 was negatively correlated with PEF. The levels of IL-4, RBC-C3bR, FEV1/FVC, and PEF were independent risk factors for the prognosis of treatment for children with moderate-to-severe asthma.

**Conclusion:**

This study demonstrated that IL-4, IL-6, and IL-12 levels in PB were associated with lung function, cellular immune function, and QOL in children with moderate-to-severe asthma.

## 1 Introduction

Asthma is a chronic respiratory disease that is usually characterized by abnormal and inflamed mucosa of the airways, as well as wheezing and shortness of breath.^[1]^ It is one of the most prevalent diseases in industrialized countries, affecting about 155 million individuals all over the world, with a reportedly increasing incidence across the developed world.^[2]^ Asthma in children is more frequent in males and may persist throughout lifetime, accompanied with clinical features such as wheezing or coughing, reversible airflow obstruction, airway dysfunction, and nocturnal awakenings. One-fourth of children with asthma have high risk of lung function loss, which attributes to exacerbation and severity of chronic asthma.^[3]^ Studies have shown that apart from interleukin (IL), mental health problems such as depression, anxiety, and behavior disorders are closely connected with the onset of asthma; thus, there is great significance in exploring the relationship among the IL level in peripheral blood (PB), the lung function of asthma children, as well as their quality of life (QOL).^[4,5]^

IL-4 serves as an essential proinflammatory cytokine in immune regulation mediated by activated T helper cells (Th) and facilitates immunoglobulin E isotype switching in B cells, growth, and differentiation of B cells and monocytes. As an important signal molecule, IL-4 can exacerbate airway inflammation through modulating eosinophils, lymphocytes, and air epithelial cells that play an important role in the pathogenesis of asthma.^[6,7]^ IL-4 is reported to play a pivotal role in phenotypic or functional changes of bronchial asthma, such as airway hyperresponsiveness, eosinophil infiltration, and mucus overproduction,^[8]^ while IL-6 is a small-size glycoprotein (21 kDa) that acts on epithelial and immune cells and is produced by cells from innate immune system, including macrophages, dendritic cells, mast cells, and neutrophils. Previous studies show that IL-6 can lead to asthma and other lung diseases via increasing airway mucus hypersecretion.^[9,10]^ IL-12 is a heterodimer made up of α and β subunits that are termed p35 and p40, respectively, and is characterized by production of interferon-γ that is also an important cytokine in antitumor immunity. It promotes antitumor immunity via activations of natural killer cells and Th1 T cells.^[11]^ A recent study has demonstrated that IL-12, an important cytokine secreted by T lymphocyte, may play an important role in the pathogenesis of asthma.^[12]^ Therefore, based on previous studies, this study is aimed to analyze the correlations between the levels of IL-4, IL-6, and IL-12 and lung function, cellular immune function, and QOL in children with moderate-to-severe asthma.

## 2 Materials and methods

### 2.1 Study subjects

Between January 2010 and December 2015, 1158 asthma children in Liaocheng People's Hospital who conformed to diagnostic criteria for asthma issued by National Pediatric Asthma Collaborative Group in 1998 were enrolled in the study and served as the experimental group. These children were diagnosed with moderate-to-severe asthma according to the staging and evaluation criteria of the disease. The study subjects consisted of 719 boys and 439 girls between the ages of 3 and 13 years, with a mean age of 6.92 ± 3.08 years. A total of 347 cases were less than 1 year in course of disease, 682 cases were 1 to 5 years, and 129 cases were more than 5 years. The exclusion criteria for patients were as follows: patients without rickets, hypoferric anemia, pneumonia, fever, fine rales in lung auscultation, patchy shadows in X-ray examination, and patients who did not take glucocorticoids within 1 month before treatment. Besides, 1075 healthy children who performed physical examination in the same period were selected as the control group (686 boys and 389 girls with a mean age of 6.68 ± 3.52 years). None of the patients had personal and family history of atopic diseases, suffered from no respiratory tract infection within a month, and did not take antihistamine drug or glucocorticoid. This study was approved by the Ethic Committee of Liaocheng People's Hospital, and informed consent was obtained from each participant and/or their legal guardians.

### 2.2 Specimen collection and testing

Each child was drawn with 5 mL fasting venous blood, and 3 mL was treated with heparin anticoagulant for testing T-cell subset and erythrocyte immunity. The remaining 2 mL blood was collected in a common test tube, and the serum was extracted after centrifugation at 3000 rpm for 10 minutes and then put into Eppendorf tubes with the capacity of 0.5 mL, marked and preserved at −80 °C for examination. Enzyme linked immunosorbent assay was adopted to detect the levels of IL-4, IL-6, and IL-12, and the reagent was purchased from Jingmei Biotech Co., Ltd., Shenzhen, China. The treatment, determination, and contend calculation of specimens were strictly conformed to kit instructions. The microplate reader was provided by Bio-Rad, Inc., Hercules, CA. Alkaline phosphatase antialkaline phosphatase was employed to test T-cell subset, and rosette formation test of erythrocyte C3b receptor was applied to examine erythrocyte immunity.

### 2.3 Treatment and therapeutic effects of asthma

The asthma children underwent conventional therapy including oxygen uptake, anti-infection and phlegm elimination after admission, and then were treated with 3 mg/kg aminophylline added with intravenous infusion of 5% glucose, in addition with slow intravenous injection of ambroxol hydrochloride (75% mg/time, dissolved in 5% glucose injection, with dose of 1 mL/time, 2 times/d [3–5 years], 1 mL/time, 3 times/d [6–10 years], and 2 mL/time, 2 times/d [over 10 years]). The asthma children were treated with several courses of treatment (a 7-day course) until significantly improved conditions.

The criteria for therapeutic effects were as follows: complete remission—absence of coughs, anhelation, and wheezes in patients’ lungs; partial remission—patients’ symptoms were mostly absent with reduced cough and anhelation as well as decreased wheezes in the lungs; no remission—patients showed no significant sign of remission in the symptoms mentioned above, or even had worse conditions regarding wheezes in lungs.

### 2.4 Lung function testing

The lung function testing was carried out by a single technologist. Related function parameters such as forced expiratory volume in 1 second (FEV1), peak expiratory flow (PEF), and FEV1/forced vital capacity (FVC) were tested via a lung function detector (Ganshorn Medizin Electronic, GmbH, Niederlauer, Germany). All testing experienced technologically satisfactory operations for at least 3 times.

### 2.5 Assessment of QOL scale

PedsQL 3.0^[13]^ (Children's Hospital and Health Center, San Diego, CA, USA) was used to assess the influence of asthma on children's life, including 28 questions (26 questions for 2–4 years parent-report questionnaire) with 4 dimensions that consisted of the symptoms of asthma (11 items), issues related to treatment (11 items), sense of anxiety (3 items), and communication (3 items). Each question was a survey in regard of occurrence frequency of a particular event within a month, and the answer options for each question were divided into 5 levels of 0 to 4 with a scale variance from 0 to 100 score. The criteria were as follows: 0 (100 points)—never, 1 (75 points)—hardly, 2 (50 points)—sometimes, 3 (25 points)—often, and 4 (0 point)—always. Each dimension score was calculated as scores of contained questions/number of answered questions, and the score of the dimension was not calculated if the answer for more than 50% questions in the dimension was assured. Total score of the scale = total scores of each question/number of answered questions in the whole scale, with higher scores signifying better QOL.

### 2.6 Statistical analysis

The SPSS 21.0 software (SPSS Inc, Chicago, IL) was applied for data analysis. Tests for normality and homogeneity test of variance were taken. Measurement data, which were consistent with normal distribution, were exhibited as mean ± standard deviation ( 
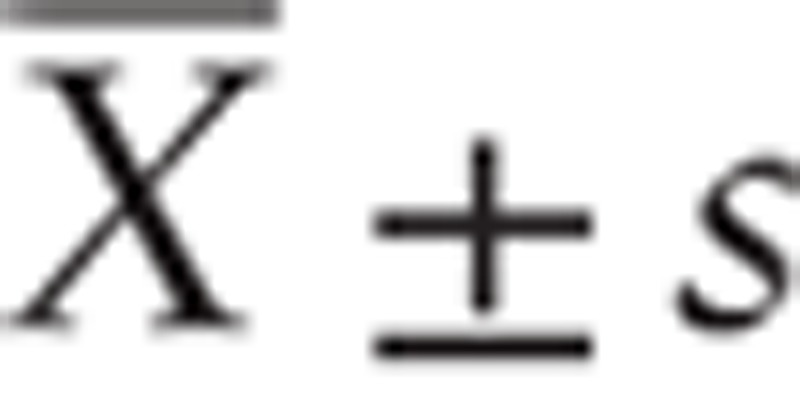
). Comparisons among groups were examined by independent sample *t* test, and comparisons before and after treatment were detected using paired *t* test. Measurement data, which were consistent of non-normal distribution, were exhibited as percentile, and comparisons of data were examined by Wilcoxon rank sum test. Enumeration data were presented as percentage or rate, with comparisons conducted by chi-square test or Fisher exact test. Pearson analysis was used to analyze the relationship of levels of IL-4, IL-6, IL-10, as well as the levels of red blood cell immune complex (RBC-IC), CD4/CD8, red blood cell C 3b receptor (RBC-C3bR), FEV1/FVC, PEF, and QOL. Logistic regression analysis was used for analyzing the related factors of the efficacy of children with moderate-to-severe asthma. The statistical significance level was set as *P* < 0.05.

## 3 Results

### 3.1 General characteristics of subjects in each group

The age, gender (male/female) ratio, as well as passive smoking proportion exhibited no significant difference between the 2 groups (all *P* > 0.05), and data revealed the comparability of the 2 groups. The IL-4 and IL-6 levels were significantly higher, while the IL-12 level was lower in the experimental group as compared to the control group (all *P* < 0.05) (Table 1).

**Table 1:**
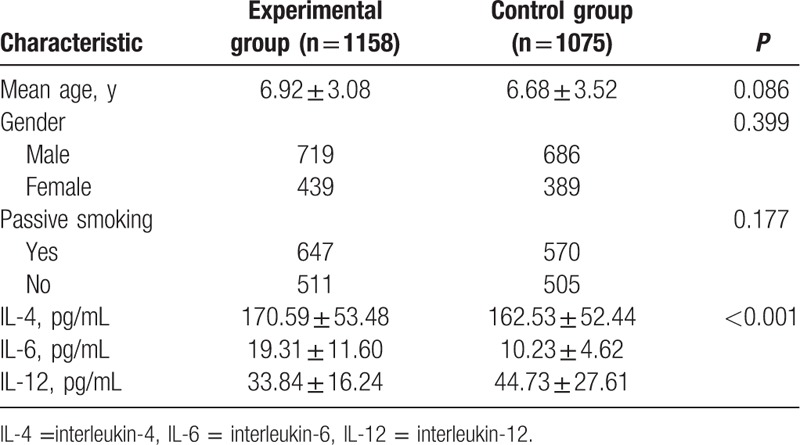
Comparisons of general characteristics of subjects in each group.

### 3.2 The levels of IL-4, IL-6, and IL-12 before and after treatment

As shown in Table 2, the IL-4 and IL-6 levels in the experimental group significantly decreased (all *P* < 0.05), but the IL-12 level remarkably increased after treatment (*P* < 0.05). The levels of IL-4, IL-6, and IL-12 in children with moderate and severe asthma were close to the levels of those in normal control group after treatment (all *P* > 0.05).

**Table 2:**
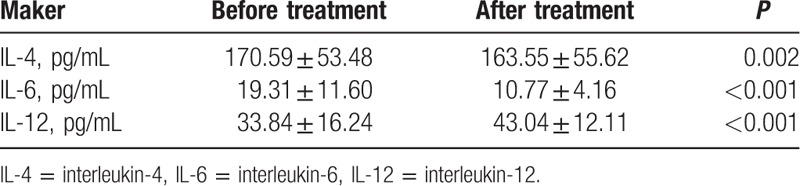
Comparison of the levels of IL-4, IL-6, and IL-12 before and after treatment.

### 3.3 Cellular immune function of children with asthma before and after treatment

The disorder of cellular immune function was significantly reduced in the experimental group after treatment, performing as elevated CD_3_, CD_4_, CD_8_, and declined CD_4_/CD_8_ in T lymphocytes. RBC-C3bR was increased and RBC-IC was reduced in erythrocyte immune when compared with those before treatment (all *P* < 0.05) (Table 3).

**Table 3:**
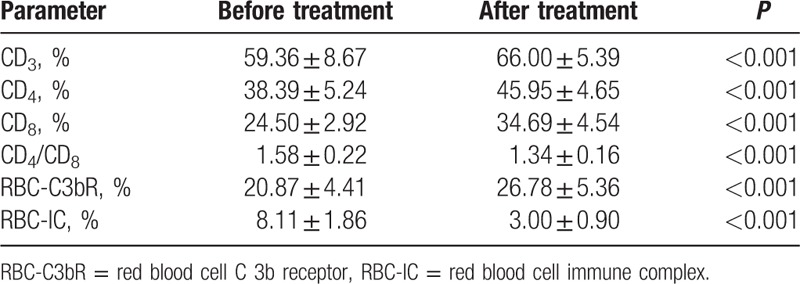
Comparisons of cellular immune function before and after treatment.

### 3.4 Comparisons of the parameters related to lung function before and after treatment

Significant differences were shown in lung function parameters including FEV1 (L), FVC (L), FEV1/FVC (%), and PEF (L/s) in experimental group before and after treatment (all *P* < 0.05). The levels of these parameters were prominently higher than those before treatment (all *P* < 0.05), suggesting that lung function in moderate- and severe-asthma children were remarkably improved after treatment (Table 4).

**Table 4:**
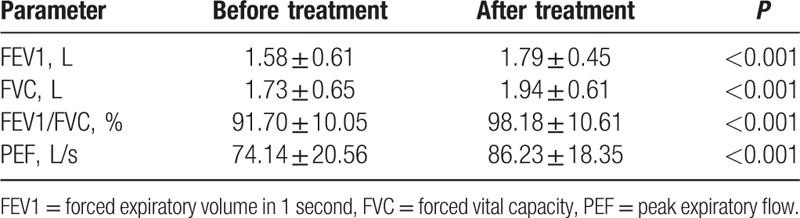
Comparisons of the parameters related to lung function before and after treatment.

### 3.5 QOL score of asthma children before and after treatment

After treatment, the symptoms of asthma in children were significantly reduced, with decreased coughs, anhelation, and wheezes in lungs, and an increased dimension score from 60.33 ± 8.80 to 87.05 ± 7.86. The children were more active and easy in using the inhaler, and the dimension score was increased to 88.64 ± 5.40 from 64.95 ± 10.09. Sense of anxiety in treatment was also significantly decreased, and the dimension score was increased to 81.97 ± 8.97 from 61.83 ± 7.79. The children were more natural and smooth in communicating with others about the understanding and expression of the disease; in this dimension, the score was increased to 87.72 ± 8.70 from 63.42 ± 7.31 (all *P* < 0.05). In general, the total score of QOL in the moderate- and severe-asthma children was 61.84 ± 9.22 before treatment with a rise to 87.66 ± 9.44 after treatment, indicating a significant difference (*P* < 0.05) (Table 5).

**Table 5:**
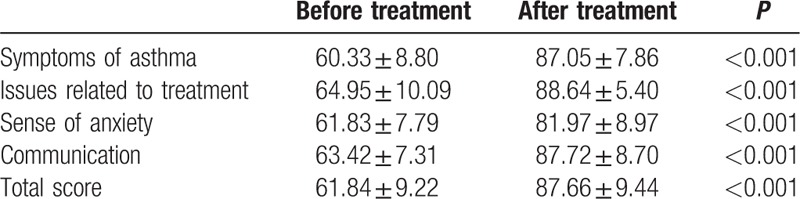
Life quality score of children with asthma before and after treatment.

### 3.6 Correlation analysis between the levels of IL-4, IL-6, and IL-12 and parameters related to cellular immune function, lung function, and QOL

The correlation analysis indicated that IL-4 was positively related to RBC-IC (*r* = 0.181, *P* < 0.001) while negatively associated with the QOL score (*r* = −0.074, *P* = 0.012), and IL-4 was independent from other parameters (*P* > 0.05). IL-6 showed negative connection with CD_4_/CD_8_, RBC-C3bR, FEV1/FVC, and QOL score (*r* = −0.153, *P* < 0.001; *r* = −0.107, *P* < 0.001; *r* = −0.068, *P* = 0.020; *r* = −0.073, *P* = 0.013), and had positive connection with PEF (*r* = 0.323, *P* < 0.001), while being independent from other parameters (*P* > 0.05). In addition, IL-12 was negatively correlated with PEF (*r* = −0.204, *P* < 0.001) but did not have connection with other parameters (*P* > 0.05) (Table 6).

**Table 6:**
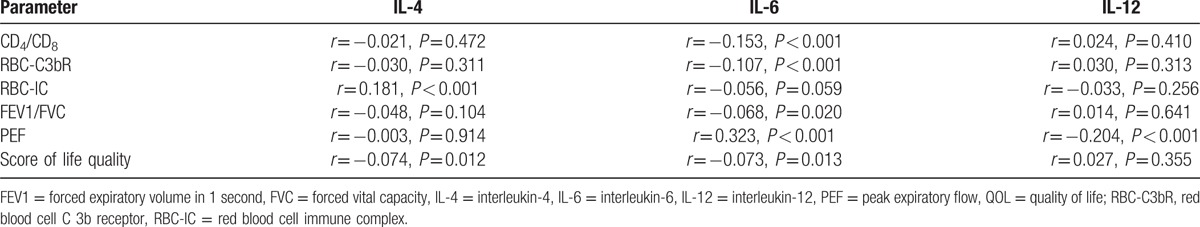
Correlation analysis among the levels of IL-4, IL-6, and IL-12 and other parameters related to cellular immune function, lung function, and QOL.

### 3.7 Logistic regression analysis for risk factors of prognosis of children with moderate-to-severe asthma

The logistic regression analysis was performed with efficacy of children as dependent variable (complete remission +  partial remission vs no remission), with factors including gender; passive smoking; levels of IL-4, IL-6, and IL-12; cellular immune function; and lung function as independent variable. The results revealed that the levels of IL-4, RBC-C3b, FEVE/FVC, and PEF were independent risk factors for prognosis of children with moderate-to-severe asthma (all *P* < 0.05) (Table 7).

**Table 7:**
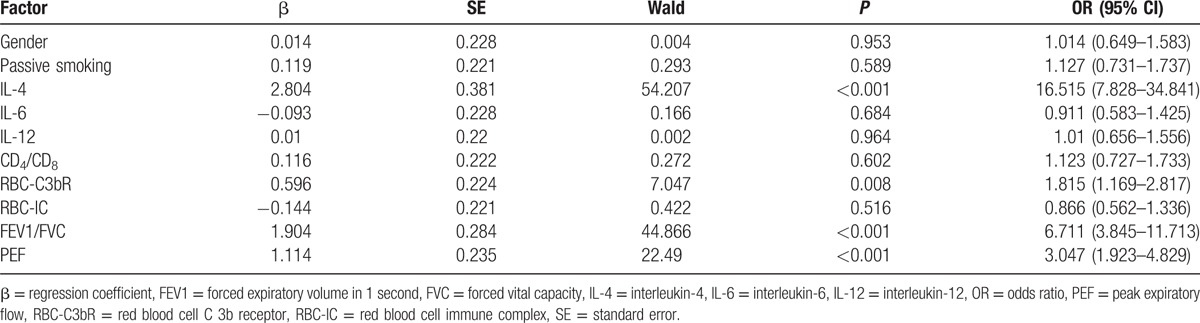
Logistic regression analysis for risk factors of prognosis of treatment for children with moderate-to-severe asthma.

## 4 Discussion

In recent years, pathogenesis of asthma has raised more and more concerns among researchers, both domestic and abroad. It is well accepted that the imbalance of cytokine network plays a vital part in asthma.^[14]^ To uncover the potential association between IL-4, IL-6, and IL-12 levels and lung function, this study investigated the roles of IL-4, IL-6, and IL-12 levels, T cell subset, and erythrocyte immune in children with moderate and severe asthma.

It is initially discovered that IL-4 and IL-6 levels were increased, whereas IL-12 level was decreased in PB of the asthma patients. IL-4, a potent activator of inflammatory responses, is considered as central Th2 cytokine of the immune system and plays an important part in fibrosis during Th2 inflammation.^[15]^ It has been recognized that IL-4 and Th2 cytokines share receptors and signal pathways, which can lead to asthma phenotype or changes in functions of bronchial asthma including airway hyperresponsiveness, eosinophil infiltration, as well as mucus overproduction of model mice.^[8]^ Th2 is overactive in asthma patients, which increases IL-4 and immunoglobulin E (IgE), stimulating the proliferation and activation of eosinophilic granulocyte, after which various inflammatory mediators are secreted, resulting in bronchial chronic inflammation and asthma.^[16]^ Emerging evidences have shown that IL-4 messenger ribonucleic acid and protein were highly expressed in airway mucosa of asthma patients; abnormal increase of IgE, promoted by IL-4, has been proved to be one of the pathogenesis of asthma, which indicates that IL-4 may induce asthma indirectly.^[17,18]^ According to a former study, IL-4 is significantly overexpressed during the acute and plateau stage of asthma, indicating that immune dysfunction participates in the development and progression of asthma.^[19]^ IL-6, produced by active macrophages and monocyte, is a multifunctional cytokine. In response to various stimuli, including allergen and respiratory viruses, IL-6 is considered to be related to tumor progression and angiogenesis.^[10]^ IL-6 may increase IL-4 during Th2 differentiation, inducing inflammation, and it may play as a potential promoting factor for asthma as well as some other lung diseases.^[20]^ Fu et al^[21]^ have reported that overexpression of IL-6 is associated with fixed airflow obstruction, and the highly expressed IL-6 is also found in bronchial epithelial cells of adults and child asthma patients. On the other hand, to the best of our knowledge, activated monocytes–macrophages, dendritic cells, as well as some other antigen-presenting cells can produce bioactive IL-12. Releasing this cytokine from antigen-presenting cells may directly lead to the differentiation of T cells into Th1 cytokine, which produces cells and acts as a costimulus of activation of effector Th1 cells during inhibition of Th2 cell generation.^[22]^ IL-12 plays as a key cytokine for the differentiation of Th0 into Th1, and the elevated IL-10 and PGE_2_ in asthma patients can promote Th2 response by the reduction of IL-12.^[23]^ It is well accepted that disproportionality of Th1/Th2 is the main cause of asthma.^[24]^ As proven, Wills-Karp^[22]^ considers that IL-12 is a potential therapy of asthma and lack of IL-12 is one of the basic potential mechanisms of atopy.

In reference, to the results of cellular immune function test, there is an improved cellular immune function in asthma patients, presenting an increase of CD_3_, CD_4_, CD_8_, and decrease of CD_4_/CD_8_ in T-cell subset, an increase of erythrocyte immune RBC-C3bR, and reduction of RBC-IC. Wang et al^[25]^ had identified that the improvement of Th immune could inhibit the airway inflammation. Iwamura et al^[26]^ had reported that the differentiation of naïve CD_4_ T cells into effector T cells is stimulated by antigen. The function of CD_8_ cells in asthma has also been demonstrated that these cells are needed for the progression of airway hyperresponsiveness after allergic sensitization, resulting in increased inflammation.^[27]^ It has been proven that the decline of long-term lung function in asthma is correlated with increase of bronchial CD_4_ and CD_8_ at baseline, and CD_3_ and CD_8_ at follow-up.^[28]^ In addition, in our study, the lung function-related parameters including FEV1 (L), FVC (L), FEV1/FVC (%), and PEF (L/s) all increased after treatment, and it is found that IL-4, IL-6, and IL-12 levels are partially correlated with lung function and QOL. The expression of IL-6 and IL-6 receptor has been proven and described in the early stages of lung development.^[29]^ It has been proven that the functional single nucleotide polymorphism of IL-6 was correlated with decline of FEV1 of smoker.^[30]^ IL-6 was identified as negatively associated with FVC.^[31]^ Wang et al^[32]^ had suggested that the IL-12 level was significantly lower in asthma patients, and the IL-12 level was positively associated with FEV1. Consequently, it is believed that IL-4, IL-6, and IL-12 levels in PB were associated with lung function and cellular immune function in children with moderate-to-severe asthma.

## 5 Conclusion

In summary, this study demonstrated that IL-4, IL-6, and IL-12 levels might be associated with the moderate-to-severe asthma of children. High levels of IL-4 and IL-6 and low levels of IL-12 were apparent in asthma patients, while IL-4, IL-6, and IL-12 might be associated with lung function and QOL of asthma patients. Many studies have been conducted on asthma before, though this study could be considered as an integrated one due to the many factors and parameters including ILs, cellular immune function, and lung function it involved. Compared to those smokers recruited in most of lung function studies, children with asthma were chosen to act as subjects in this study, which yielded less-confounding factors than an adult study. With improved understanding on the correlation of these factors and asthma, we are hopefully able to provide novel treatment and elevate the QOL of asthma patients by limiting the levels of IL-4 and IL-6, and improving the expression of IL-12, through which the symptoms of asthma might be alleviated. However, an experimental result has been shown here, though the underlying mechanism of these ILs in asthma remains unclear, and in the QOL testing of our study, questionnaires were used, of which data remain inaccurate. We suggest more prospective studies to investigate the precise interaction among IL-4, IL-6, and IL-12 performed to develop new therapeutic strategies for the treatment of bronchial asthma and relieving airway inflammation.

## Acknowledgments

We thank the reviewers for critical comments.
